# Supplementary material for: Lessons learned and lessons missed: impact of the coronavirus disease 2019 (COVID-19) pandemic on all-cause mortality in 40 industrialised countries and US states prior to mass vaccination
Source: Wellcome Open Res. 2022 Feb 15;6:279. Originally published 2021 Oct 18. [Version 2] doi: 10.12688/wellcomeopenres.17253.2 (PMC8861471; doi:10.12688/wellcomeopenres.17253.2)
Supplement: Peer review history

# Peer review: Reviewer response for version 2

**DOI:** 10.21956/wellcomeopenres.19577.r48703

**Published:** 2022-02-21

**Authors:** Rajeev Gupta

**Affiliations:** 1 Academic Research Development Unit, Rajasthan University of Health Sciences, Jaipur, Rajasthan, India; 2 Eternal Heart Care Centre and Research Institute, Jaipur, India

The article is now acceptable for indexing.

Is the work clearly and accurately presented and does it cite the current literature?

Partly

If applicable, is the statistical analysis and its interpretation appropriate?

I cannot comment. A qualified statistician is required.

Are all the source data underlying the results available to ensure full reproducibility?

Yes

Is the study design appropriate and is the work technically sound?

Yes

Are the conclusions drawn adequately supported by the results?

Yes

Are sufficient details of methods and analysis provided to allow replication by others?

Yes

Reviewer Expertise:

Cardiovascular epidemiology

I confirm that I have read this submission and believe that I have an appropriate level of expertise to confirm that it is of an acceptable scientific standard.

# Peer review: Reviewer response for version 2

**DOI:** 10.21956/wellcomeopenres.19577.r48702

**Published:** 2022-02-17

**Authors:** Virgilio Gómez-Rubio

**Affiliations:** 1 Department of Mathematics, School of Industrial Engineering-Albacete, Universidad de Castilla-La Mancha, Ciudad Real, Spain

Thanks for your reply and the changes in the manuscript that address all my concerns raised in my previous review.

Is the work clearly and accurately presented and does it cite the current literature?

Yes

If applicable, is the statistical analysis and its interpretation appropriate?

Partly

Are all the source data underlying the results available to ensure full reproducibility?

Yes

Is the study design appropriate and is the work technically sound?

Yes

Are the conclusions drawn adequately supported by the results?

Yes

Are sufficient details of methods and analysis provided to allow replication by others?

Yes

Reviewer Expertise:

Statistics

I confirm that I have read this submission and believe that I have an appropriate level of expertise to confirm that it is of an acceptable scientific standard.

# Peer review: Reviewer response for version 1

**DOI:** 10.21956/wellcomeopenres.19069.r47314

**Published:** 2022-01-06

**Authors:** Virgilio Gómez-Rubio

**Affiliations:** 1 Department of Mathematics, School of Industrial Engineering-Albacete, Universidad de Castilla-La Mancha, Ciudad Real, Spain

The authors have presented an interesting analysis of the excess mortality in 40 countries during the first year of the COVID-19 pandemic. They have used publicly available data and Bayesian hierarchical models for the analysis. They have also provided links to the data sources as well as a link to a GitHub repository with the R code and datasets to make sure that the results can be replicated. As part of the analysis, they conduct a validation of the model which makes me think that the results obtained (and their conclusions) are reliable. The conclusions are supported by the data and the analysis carried out.

 My only concern is about the "model averaging approach" followed by the authors. First of all, why not select a single model using the different model selection criteria available in INLA? Some of the models are nested so it makes me wonder whether the 'extra' components are needed. Most likely the answer is yes, and then the authors should choose the model with the extra components.

 Secondly, I agree that following a Bayesian model averaging is useful but I am not sure that this can be achieved by simply "pooling" the samples obtained from INLA (see, for example, Gómez-Rubio et al., 2020 ^1^ where we illustrate how to do BMA with INLA by weighting the different models according to the marginal likelihood). The authors should have weighted the samples according to the values of the marginal likelihood for each model (which are reported by INLA). These values could have been used to compute the posterior probability of each model for model selection as well. Having said this, it looks like the results produced are similar to those provided by other analyses, which may indicate that weighting the samples have no major impact (i.e., all models produce very similar death estimates), but I think this is something that the authors should check.

Is the work clearly and accurately presented and does it cite the current literature?

Yes

If applicable, is the statistical analysis and its interpretation appropriate?

Partly

Are all the source data underlying the results available to ensure full reproducibility?

Yes

Is the study design appropriate and is the work technically sound?

Yes

Are the conclusions drawn adequately supported by the results?

Yes

Are sufficient details of methods and analysis provided to allow replication by others?

Yes

Reviewer Expertise:

Statistics

I confirm that I have read this submission and believe that I have an appropriate level of expertise to confirm that it is of an acceptable scientific standard, however I have significant reservations, as outlined above.

## References

[1] : Bayesian Model Averaging with the Integrated Nested Laplace Approximation. *Econometrics* .2020;8(2) : 10.3390/econometrics8020023 10.3390/econometrics8020023

# Response

**Published:** 2022-02-01

**Authors:** Majid Ezzati

**My only concern is about the "model averaging approach" followed by the authors. First of all, why not select a single model using the different model selection criteria available in INLA? Some of the models are nested so it makes me wonder whether the 'extra' components are needed. Most likely the answer is yes, and then the authors should choose the model with the extra components.**

 This is a good point. We used a model ensemble and model averaging approach for three reasons: First, we have shown in prior work ^1^ that for forecasts and projections, which by definition involve out-of-sample estimation, a model ensemble/average outperforms the best available model when the projection timeline is longer than a few time units (which would be a few weeks here) – see Appendix Figure 3 in Kontis *et al.* ^1^. This is consistent with findings from other works that involve forecasting and projections ^2-4^. Second, which single model performs best in out-of-sample prediction depends on the country and year, even among those that have related terms, as seen in Appendix Figure 1 of Kontis *et al.*. ^1^. This year-to-year and country-to-country variability in what the best model is, is itself due to the complex behaviour of mortality data over space and time, and means that the best model for the pandemic period is uncertain. In our validation analyses, 13 out of 16 models ranked best (i.e., had the smallest absolute projection error) for at least one country and one validation year. Even when looking at individual countries, in only 2 out of 33 countries did the same model emerge as the best model in all three validation years. What model is best is not only about choosing the one with the most components, but also the choice of prior (e.g., the order of the autoregressive term). Finally, and as a consequence of the aforementioned issues, the use of multiple models and model averaging provides a more complete picture of the inherent uncertainty in how many excess deaths the pandemic has caused than approaches that are not probabilistic or use a single model.

**Secondly, I agree that following a Bayesian model averaging is useful but I am not sure that this can be achieved by simply "pooling" the samples obtained from INLA (see, for example, Gómez-Rubio et al., 2020 where we illustrate how to do BMA with INLA by weighting the different models according to the marginal likelihood). The authors should have weighted the samples according to the values of the marginal likelihood for each model (which are reported by INLA). These values could have been used to compute the posterior probability of each model for model selection as well. Having said this, it looks like the results produced are similar to those provided by other analyses, which may indicate that weighting the samples have no major impact (i.e., all models produce very similar death estimates), but I think this is something that the authors should check.**

 Although in-sample measures of fit are well-suited for applications where there is an interest in the parameters of the model, for projection tasks like ours, models must be evaluated and weighted based on their out-of-sample performance, which is what they have been designed for. Projections of individual models can be combined in a fully Bayesian approach, by assigning weights based on out-of-sample performance, or simply by assigning uniform weights (i.e., using an unweighted average). In this work, we used the latter approach for the following reasons: First, methods for combing projections in a fully Bayesian manner typically use simpler base models (e.g. simple linear regressions) ^4,5^ and in our case, such an approach would not have been feasible due to its computational complexity. Second, calculating weights based on out-of-sample performance requires further validation to decide how weights should be defined (e.g., as the reciprocal of absolute projection error, or in some other way that penalises models with larger errors), as we have done in prior work ^1^. Validating the choice of model weights in turn requires an additional hold-out period which in our case was not available for many countries, due to the short duration of their time series. Choosing uniform model weights avoids this problem and has been shown to produce equal or superior results to using non-equal weights in many applications ^2,6^.

 Nonetheless, based on this comment, we now include a sensitivity analysis on how the different models are weighted. Specifically, in the sensitivity analysis, the number of draws from each model was inversely proportional to the absolute error of prediction in the validation analyses. As seen, the results are robust to this choice, with the current approach having the advantage of being usable for countries with shorter time series.

**References**

 1. Kontis V, Bennett JE, Mathers CD, Li G, Foreman K, Ezzati M. Future life expectancy in 35 industrialised countries: projections with a Bayesian model ensemble. *Lancet* 2017; **389**(10076): 1323-35.

 2. Clemen RT. Combining forecasts: A review and annotated bibliography. *International Journal of Forecasting* 1989; **5**(4): 559-83.

 3. Makridakis S, Winkler RL. Averages of Forecasts: Some Empirical Results. *Management Science* 1983; **29**(9): 987-96.

 4. Raftery AE, Gneiting T, Balabdaoui F, Polakowski M. Using Bayesian Model Averaging to Calibrate Forecast Ensembles. *Monthly Weather Review* 2005; **133**(5): 1155-74.

 5. Eklund J, Karlsson S. Forecast Combination and Model Averaging Using Predictive Measures. *Econometric Reviews* 2007; **26**(2-4): 329-63.

 6. Timmermann A. Chapter 4 Forecast Combinations. In: Elliott G, Granger CWJ, Timmermann A, eds. Handbook of Economic Forecasting: Elsevier; 2006: 135-96.

# Peer review: Reviewer response for version 1

**DOI:** 10.21956/wellcomeopenres.19069.r46937

**Published:** 2021-11-18

**Authors:** Rajeev Gupta

**Affiliations:** 1 Academic Research Development Unit, Rajasthan University of Health Sciences, Jaipur, Rajasthan, India; 2 Eternal Heart Care Centre and Research Institute, Jaipur, India

**General comments:**

1. This is an interesting article on the spread of COVID-19 in 40 industrialized countries. However, the article has limited information on the lessons learned from these studies. This is the major weakness of the study. Qualitative studies should be mentioned.
2. Many books have been written on the topic and it would be useful for the authors to look at qualitative information from some of them. I would specifically mention the following (which I have read (sic) and many others that I could not):
  1. Michael Lewis. The premonition: a pandemic story.
  2. Niall Ferguson: Doom: the politics of catastrophe.
  3. Jeremy Farrar and Anjana Ahuja. Spike: The virus vs the people, the inside story.
  4. Adam Kucharaski. The rules of contagion.
  5. Many journal as well as newspaper articles also provide insights into the failures and successes.
3. Would it be worthwhile to add the delta wave epidemic that swept through India, UK and US and is currently creating havoc in Europe?

**Specific comments:**

**Abstract:**

1. Data from US and US states have been prominently displayed in the Results section but are missing.
2.  The conclusion statement does not mention the lessons missed (NPI, delays, etc).

**Methods:**

1. Reference to the “Our World in Data” website is not provided.
2. The authors have extensively adjusted for the temperature variation data. This is important as this is an important determinant. Two other variables could be important: (a) Age distribution of populations in these countries; and (b) Ambient pollution. Data of both are widely available and it would be interesting to evaluate whether these variables influence the outcomes.
3. Page 7, para 2, line 2: is it mid-February 2021? Please correct.

**Results:**

1. Do we have data on excess mortality for US states? May be interesting to add (unless it is part of a forthcoming study).
2. No data are presented regarding “lessons learned”. May be useful to study the social determinants such as GDPs, income, and health systems.

**Discussion:**

1. Please discuss the data presented and their limitations, and then focus on the implications of the study.

Is the work clearly and accurately presented and does it cite the current literature?

Partly

If applicable, is the statistical analysis and its interpretation appropriate?

I cannot comment. A qualified statistician is required.

Are all the source data underlying the results available to ensure full reproducibility?

Yes

Is the study design appropriate and is the work technically sound?

Yes

Are the conclusions drawn adequately supported by the results?

Yes

Are sufficient details of methods and analysis provided to allow replication by others?

Yes

Reviewer Expertise:

Cardiovascular epidemiology

I confirm that I have read this submission and believe that I have an appropriate level of expertise to confirm that it is of an acceptable scientific standard, however I have significant reservations, as outlined above.

# Response

**Published:** 2022-02-01

**Authors:** Majid Ezzati

**1. This is an interesting article on the spread of COVID-19 in 40 industrialized countries. However, the article has limited information on the lessons learned from these studies. This is the major weakness of the study. Qualitative studies should be mentioned.**

 The revised paper refers to over 25 qualitative and evidence synthesis studies, either across countries or for specific countries.

**2. Many books have been written on the topic and it would be useful for the authors to look at qualitative information from some of them. I would specifically mention the following (which I have read (sic) and many others that I could not):**

  

1. **Michael Lewis. The premonition: a pandemic story.**
2. **Niall Ferguson: Doom: the politics of catastrophe.**
3. **Jeremy Farrar and Anjana Ahuja. Spike: The virus vs the people, the inside story.**
4. **Adam Kucharaski. The rules of contagion.**
5. **Many journal as well as newspaper articles also provide insights into the failures and successes.**

We are aware of the large number of books, and the even larger number of newspaper articles and opinion pieces, on the COVID-19 pandemic. We have cited some key ones that are directly related to our paper including books as well as newspaper articles.

**3. Would it be worthwhile to add the delta wave epidemic that swept through India, UK and US and is currently creating havoc in Europe?**

 We believe that the Delta and Omicron waves should be the subject of distinct analyses for two reasons: First, as stated in the Introduction of our paper, the effectiveness of vaccination in reducing deaths motivates distinct analyses and interpretation prior to v versus after mass vaccination, with the focus of our paper on the former. Second, because the number of deaths that would be expected had the pandemic not occurred is not directly measurable, its estimation requires projection modelling based on past time-series of weekly deaths. The longer the period of projection since the beginning of the pandemic, the more difficult and uncertain the projections, possibly requiring methods that are different from those used for the first year.

**Specific comments:**

**Abstract:**

**Data from US and US states have been prominently displayed in the Results section but are missing.**

 We have included in the abstract as correctly suggested.

**The conclusion statement does not mention the lessons missed (NPI, delays, etc).**

 We have made the use of NPIs explicit in the abstract’s conclusion, from its original implicit use.

**Methods:**

**Reference to the “Our World in Data” website is not provided.**

 We had initially done this as a hyperlink. We have now also cited the corresponding publication.

**The authors have extensively adjusted for the temperature variation data. This is important as this is an important determinant. Two other variables could be important: (a) Age distribution of populations in these countries; and (b) Ambient pollution. Data of both are widely available and it would be interesting to evaluate whether these variables influence the outcomes.**

 Our statistical models were designed to estimate weekly death rates, had the pandemic not occurred. With this objective, the models do not adjust for temperature or any other variable. Rather the models include, as covariates, variables that are predictors of mortality, vary from week to week (because those that do not vary from week to week are captured by other model terms), and are themselves not affected by the pandemic (so that we make unbiased estimates of counterfactual, no-pandemic mortality).

 Temperature was included in our model because it is an established predictor of short-term mortality, and was itself not affected by the pandemic. We did not include air pollution in the model, because the pandemic and the associated policy and behavioural responses, in particular the reduction in vehicular traffic and industrial activity, led to changes in air pollution ^1,2^. This in turn means that the observed pollution is different from what pollution levels would have been without the pandemic. In contrast, researchers have estimated a short-term pandemic impact of only ~0.01°C on ambient temperature ^3^, which is orders of magnitude smaller than seasonal and interannual variation.

 The age distribution of each country is already embedded in past data on deaths and population, and is therefore implicitly used to produce our projections. More details provided below.

 As the Reviewer correctly points out, our main results are based on analysis that used data from all ages together, for three reasons: First, at least three terms in our model implicitly take into account any change in age distribution that might occur from week to week, which is the relevant time interval for our work: (1) The model has a random intercept for each week of the year (θ _week_) which allows deaths to change from week to week, some of which may be partly due to differential changes in age groups; the weekly terms repeat across years although their magnitude can change due to variations in temperature. (2) The autoregressive structure of the model allows these terms to be related to deaths in the previous week which means that phenomena that gradually change the age structure of deaths on a weekly scale (e.g., weekly dynamics of flu) are captured. (3) Similarly, we have a term that captures monthly mortality, also with autoregressive structure at annual scale, which implicitly captures phenomena that change deaths at annual scale in a correlated way. Second, when the time unit of analysis is the week, rather than the year as is the case in most mortality projections, the number of deaths in many country-week-age units is zero or small. Using all ages together can make model fits more stable, hence trading off age-related precision with how well model parameters are estimated. Third, running a single model for all ages means that the uncertainty of the estimates is correctly reported.

 That being said, we have also repeated the analysis by age group and compare the estimates with those of the combined model in the section titled *statistical methods*. This comparison shows that the two approaches generate similar estimates of counterfactual no-pandemic mortality, for the aforementioned reasons.

**Page 7, para 2, line 2: is it mid-February 2021? Please correct.**

 Assuming the comment refers to the sentence starting with “We used data on weekly deaths from the start of time series through mid-February 2020 …”, “mid-February 2020” is correct as estimation of model parameters was based on data before the pandemic.

**Results:**

**Do we have data on excess mortality for US states? May be interesting to add (unless it is part of a forthcoming study).**

 Figures 2, 4 and 8, and the associated text, present results on excess mortality for US states.

**No data are presented regarding “lessons learned”. May be useful to study the social determinants such as GDPs, income, and health systems.**

 The countries studied here are all high-income although they differ in health care spending and health service infrastructure ^4^. It would be a separate body of work, and a distinct paper, to quantify how various features of health system – from local public health and primary care to specialist care – may have affected the outcome of the pandemic. We now discuss this issue at the beginning of the Discussion section, with reference to a number of studies that have qualitatively and quantitative analysed the role of both health system resilience and early social distancing measures on the impacts of pandemic.

**Discussion:**

**Please discuss the data presented and their limitations, and then focus on the implications of the study.**

 We have reorganised the text as suggested.

**References**

 1. Venter ZS, Aunan K, Chowdhury S, Lelieveld J. COVID-19 lockdowns cause global air pollution declines. *Proc Natl Acad Sci U S A* 2020; **117**(32): 18984-90.

 2. Liu F, Page A, Strode SA, et al. Abrupt decline in tropospheric nitrogen dioxide over China after the outbreak of COVID-19. *Sci Adv* 2020; **6**(28): eabc2992.

 3. Forster PM, Forster HI, Evans MJ, et al. Current and future global climate impacts resulting from COVID-19. *Nature Climate Change* 2020; **10**(10): 913-9.

 4. Haldane V, De Foo C, Abdalla SM, et al. Health systems resilience in managing the COVID-19 pandemic: lessons from 28 countries. *Nat Med* 2021; **27**(6): 964-80.
